# Causes de décès à Dakar et politique de santé

**DOI:** 10.11604/pamj.2019.32.187.10333

**Published:** 2019-04-17

**Authors:** Mohamed Maniboliot Soumah, Mariame Koumare, Mor Ndiaye, Mamadou Lamine Sow

**Affiliations:** 1 Service de Médecine Légale et Médecine du Travail, Faculté de Médecine, de Pharmacie et d'Odontologie, Université Cheikh Anta Diop, Dakar, Sénégal

**Keywords:** Mortalité, traumatisme, prévention, Mortality, trauma, prevention

## Abstract

L'étude de la mortalité permet d'identifier les principaux problèmes de santé publique des pays dont ils émanent. Elle donne une idée des mesures préventives et médico-curatives et des investissements dans la recherche qui pourraient accroître l'espérance de vie de la population. L'objectif général de ce travail était d'évaluer les causes de mort dans la population générale à partir des informations figurant sur les registres de décès à Dakar. Les objectifs spécifiques étaient de déterminer les causes de mort, d'identifier les facteurs sociodémographiques influençant les décès et d'identifier les genres de mort et type de mort. Il s'agit d'une étude rétrospective portant sur les cas de décès dans la population générale de Dakar, de 2003 à 2012, ayant bénéficié d'une autopsie. Les sources des données étaient les registres des autopsies de l'Hôpital Aristide le Dantec (HALD) et de l'Hôpital Général de Grand-Yoff (HOGGY), seuls hôpitaux de Dakar où sont effectuées des autopsies. Les informations contenues dans les différentes sources de données ont été rapportées sur une fiche d'enquête analysée par Epi-info version 6.04. Durant la période d'étude, nous trouvions 985 décès parmi lesquels, 693 morts violentes (70,3%), 261 morts naturelles (26,5%) et 14 morts indéterminées (1,4%). Il y avait une prédominance des accidents de la circulation au nombre de 394 (40%). Concernant le genre de mort, plus de la moitié des cas de décès étaient accidentels donc des morts violentes. Les décès par accident de la circulation se passaient le plus souvent en zone préhospitalière ou à l'arrivée à l'hôpital. Le ministère de la santé devra continuer les efforts sur la prévention et la prise en charge des maladies transmissibles. Les deux priorités devront être les maladies cardiovasculaires et les traumatismes.

## Abstract (en)

The study of mortality enables to identify the major public health problems in the countries under consideration. It can give an indication on the preventive and “medico-curative” measures as well as on the investments in research that could increase life expectancy of the population. This study aims to evaluate the causes of death in the general population based on the informations contained in the register of deaths in Dakar. Our specific aim was to determine the causes of death, to identify the socio-demographic factors influencing death and to identify the kinds of death and types of death. We conducted a retrospective study on the cases of death in the general population who underwent an autopsy in Dakar, from 2003 to 2012. Data were taken from the autopsy registers from the Aristide Le Dantec Hospital (HALD) and the General Hospital of Grand Yoff (HOGGY), which are the only hospitals in Dakar where autopsies are made. The informations contained in the different data sources were recorded on a survey form and analyzed using Epi-Info 6.04. During the study period, 985 patients died among whom 693 of violent death (70.3%), 261 of natural death (26.5%), and 14 of unspecified cause (1.4%). There was a predominance of traffic accidents (394; 40%). With respect to the kind of death, more than half of the cases were due to traffic accidents (violent deaths). Traffic-related deaths most often occurred during the pre-hospitalization period or on arrival to the hospital. The Ministry of Health should continue to promote measures for preventing and managing communicable diseases. The two priorities should be as follows: cardiovascular diseases and traumas.

## Introduction

La mortalité est le rapport entre le nombre de décès et l'effectif moyen de la population dans un lieu donné pendant une période déterminée. Elle permet d'avoir une approche scientifique pour élaborer un plan de développement d'où l'intérêt de ce sujet. Les indicateurs de mortalité évitable ont été définis à partir des listes de causes de décès et de classes d´âges, généralement sélectionnées sur la base de la littérature et les dires des experts. En France, la mortalité évitable cible plus particulièrement la mortalité liée aux comportements à risque et à la prévention primaire. D´autres, souvent utilisées dans les pays anglo-saxons, sélectionnent des causes dont l´éventualité est plus directement associée à un acte médical (prévention secondaire, soins…). Ces différents choix ont pour objectif d´identifier les principaux problèmes de santé publique des pays dont ils émanent. Ce suivi passe par la connaissance systématique de tous les décès par le corps des médecins. Le certificat de décès a un rôle juridique mais aussi épidémiologique car il permet l´établissement de la statistique de santé publique. Au Sénégal, le taux de mortalité semble être encore élevé, les causes de décès revêtent une grande importance dans l'évaluation de l'état de la santé de la population et de la qualité des soins. Elles donnent une idée des mesures préventives et médico-curatives et des investissements dans la recherche qui pourraient accroître l'espérance de vie de la population. Ce sont les résultats des statistiques sur les causes médicales de décès qui mettent ainsi en évidence certaines caractéristiques épidémiologiques importantes. La connaissance des causes de décès contribue à l´évaluation et au suivi des actions de santé publique. C'est pourquoi l'objectif général de ce travail était d'évaluer les causes de mort dans la population générale à partir des informations figurant sur les registres de décès à Dakar. Les objectifs spécifiques étaient de déterminer les causes de mort, d'identifier les facteurs sociodémographiques influençant les décès et d'identifier les genres de mort et type de mort.

## Méthodes

Il s'agit d'une étude rétrospective portant sur les cas de décès ayant bénéficié d'une autopsie dans la population générale de Dakar, de 2003 à 2012. Les sources des données étaient les registres des autopsies de l'Hôpital Aristide le Dantec (HALD) et de l'Hôpital Général de Grand-Yoff (HOGGY), seuls hôpitaux de Dakar où sont effectuées des autopsies. Le rapport d'autopsie et le certificat de genre de mort nous ont permis d'avoir des informations sur les circonstances de décès, du type de mort, des causes de décès. Les critères recherchés étaient l'âge, le sexe, adresse (Dakar centre, Dakar périphérie, banlieue, autres), lieu de décès, type de mort (naturelle, suspecte, violente, toxique, subite), les causes de décès, le genre de mort (naturelle, homicide, accident, suicide). Les informations contenues dans les différentes sources de données ont été rapportées sur une fiche d'enquête analysée par Epi-info version 6.04. Les variables qualitatives ont fait l'objet d'une analyse descriptive en pourcentage. Les variables quantitatives ont été analysées à l'aide de moyenne, de médiane et d'écart-type.

## Résultats

**Figure 1:**
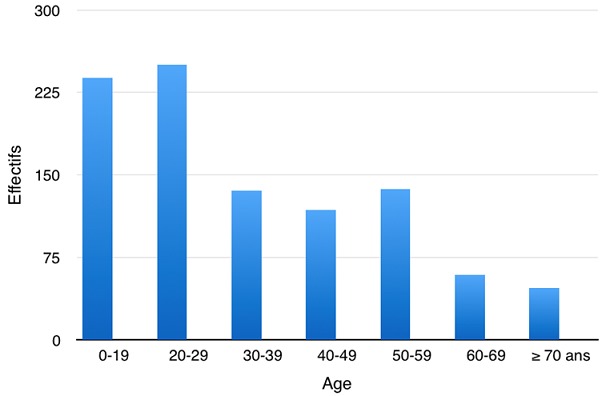
Répartition des sujets décédés selon l'âge

**Figure 2:**
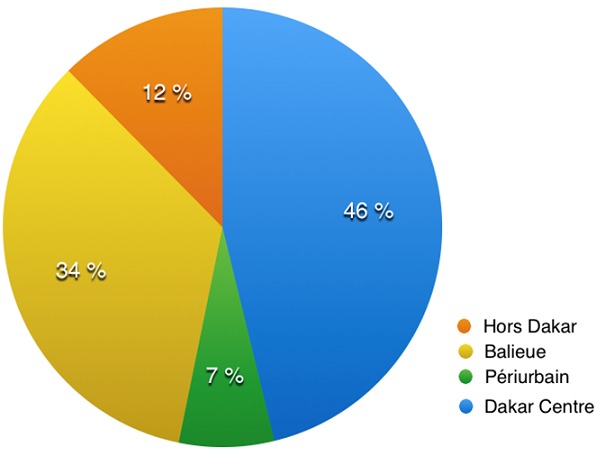
Répartition des décès selon le lieu de residence

Durant la période d'étude, nous avions trouvé 985 décès parmi lesquels, 693 morts violentes (70,3%), 261 morts naturelles (26,5%) et 14 morts indéterminées (1,4%). Il y avait une prédominance des accidents de la circulation au nombre de 394 (40%). La voie publique était le lieu de décès de 594 personnes de la population d'étude (60,3%). La moyenne d'âge des sujets décédés était de 34 ans avec des extrêmes allant de 0 à 90 ans (Figure 1). La classe d'âge la plus touchée était celle des 20-29 ans (n = 250 soit 25,4%). Les décès étaient à prédominance masculine avec 846 cas (85,9%). Selon leurs adresses, 46,2% des patients venaient du Centre de Dakar et environ 1 sujet sur 3 habitait la banlieue de Dakar (Figure 2).

**Figure 3:**
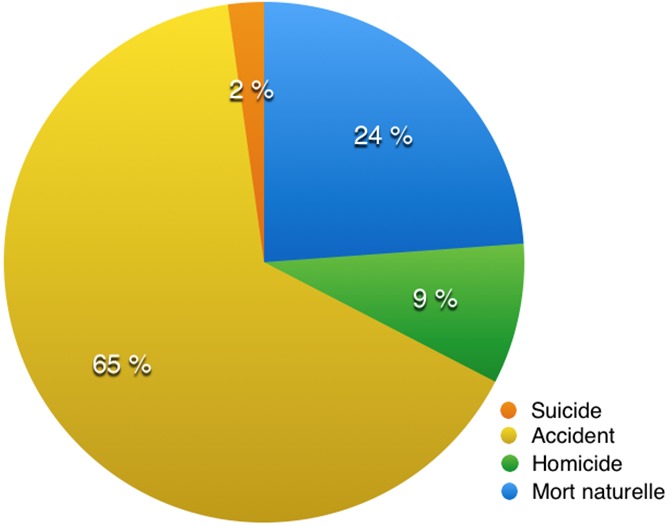
Répartition des décès selon le genre de mort

Les accidents de circulation étaient la première cause de décès des patients de la population d'étude avec 40% (Tableau 1). Les autres causes de décès étaient des morts violentes avec les homicides par coups et blessures (n = 86), les noyades (n = 54), les pendaisons (n = 20), les strangulations (n = 12), les brulures (n = 38) et les chutes (n = 52). Concernant le genre de mort (Figure 3), plus de la moitié des cas de décès étaient accidentels (n = 621 soit 65,1%) donc des morts violentes.

**Tableau 1:** Répartition des patients en fonction des causes de décès

Causes	Effectifs	Pourcentage
Maladies infectieuses et parasitaires	17	1,7
Tumeurs	4	0,4
Accidents vasculaires cérébraux	10	1
Cardiopathies ischémiques	143	14,5
Appareil respiratoire	35	3,5
Appareil digestif	26	2,6
Accident de la circulation	394	40
Accident de travail	32	3,2
Accident domestique	62	6,3
Autres	262	26,5
**Total**	**985**	**100**

S'agissant des types de mort, les morts subites (1,2%), les morts toxiques (1,2%) et les morts suspectes (0,8%) étaient peu retrouvées. Nous avions retrouvé surtout les morts violentes (70,3%) et les morts naturelles (26,5%). Nous avions trouvé une corrélation significative entre l'âge et la cause de décès (p = 0,0001), les affections cardiovasculaires étant plus fréquentes entre 50 et 59 ans (Tableau 2). Les accidents de la circulation concernaient surtout les adolescents entre 10 et 19 ans. Cette classe d'âge était aussi touchée par les autres causes de mort violente, suivie de la classe d'âge entre 20 et 29 ans. Nous n'avions pas retrouvé de corrélation significative entre le sexe et les causes de décès (p = 0,501).

**Tableau 2:** Répartition des causes de décès en fonction de l’âge

Tranche d’âge Cause	0-19	20-29	30-39	40-49	50-59	60-69	70-100	Total
Maladies infectieuses	2	2	5	3	2	0	3	17
Tumeurs	0	0	0	0	2	1	1	4
Maladies cérébro-vasculaires	1	1	2	3	3	0	0	10
Cardiopathie ischémique	9	20	12	24	53	15	10	143
Appareil respiratoire	7	3	5	8	8	2	2	35
Appareil digestif	1	4	8	5	4	2	2	26
Accident de la circulation	118	98	51	43	41	23	20	394
Accident de travail	0	20	6	4	2	0	0	32
Accident domestique	28	16	4	4	4	5	1	62
Autres	71	86	44	24	17	11	9	262
Total	237	250	137	118	136	59	48	985

Les décès par accident de la circulation se passaient le plus souvent en zone préhospitalière ou à l'arrivée à l'hôpital. Les décès par cardiopathie ischémique se passaient surtout au domicile. Nous n'avions pas trouvé de corrélation significative entre les causes de décès et le lieu de résidence (p = 0,1).

## Discussion

Les limites de notre étude sont liées à la nature rétrospective de l'étude. Certaines informations concernant les décès par accident de circulation ou par coups et blessures n'étaient pas recueillies. Certaines causes de décès étaient indéterminées et quelques cas d'autopsie impraticable. Nous avons évalué la fréquence des causes de décès recueillies dans les registres des morgues des hôpitaux (HALD, HOGGY) représentatifs de Dakar. Elle varie d'un pays à l'autre dans le monde. Ces informations assurent une couverture des évènements et leur datation précise. Dans notre étude les décès violents étaient plus représentés dans les 2 sites soit 70,3%. Concernant les causes de décès, elles sont dans l'ensemble mal connues en Afrique sub-saharienne [1]. Cette situation est due au fait que des décès ont lieu sans qu'un médecin n'ait vu le malade avant sa mort ou qu'une autopsie n'ait pu être faite après. La méthode d'enquête utilisée ici a permis de déterminer pour la plupart des décès s'ils étaient dus à une cause violente ou non.

Nous avions une prédominance masculine parmi la population de notre échantillon avec 86% d'homme et 14% de femmes soit un sexe ratio 6,1. La tranche d'âge la plus représentée était comprise entre 20 et 29 ans et était victime d'accidents et mort violentes. La majorité des accidents de la circulation concernaient des victimes entre 0 et 19 ans. Les cardiopathies ischémiques étaient plus importantes entre 50 et 59 ans. L'accident de circulation était de prédominance masculine. Ces résultats sont comparables à ceux de Naumann *et al.* [2]. La surmortalité masculine est présente à tous les âges, mais surtout chez les jeunes (15 à 35 ans). Le phénomène est plus accentué en Afrique avec la constitution jeune des populations, avec une pyramide des âges élargie à la base et une faible espérance de vie [3]. La plupart des pays en développement, en revanche doivent estimer le nombre de décès selon les causes à partir de données lacunaires. Des progrès dans ce domaine sont nécessaires pour améliorer la santé et réduire les décès. La comptabilisation annuelle des décès et la détermination des causes sont essentielles pour évaluer l'efficacité du système de santé d'un pays, que l'on estime aussi en mesurant l'impact des maladies et des traumatismes. Ces chiffres permettent aux autorités sanitaires de déterminer si elles prennent des mesures de santé publique adéquates.

Au Sénégal et dans les pays en voie de développement, les traumatismes sont négligés dans les statistiques nationales et nous restons au concept de primauté des maladies transmissibles. La place de plus en plus importante des traumatismes et des violences doit faire réfléchir sur les priorités en santé dans nos pays. « Pourquoi continuons-nous à mettre autant de ressources sur la recherche sur l'infection à VIH que dans les causes de suicide et dans la prévention des accidents de la circulation ? » Telle est la question posée déjà en 1997 dans l'éditorial du Lancet (Vol 349, May 3, 1997). En effet les principales causes de décès étaient en 1990 les cardiopathies ischémiques (6,3 millions), les accidents vasculaires cérébraux (4,4 millions), les infections respiratoires basses (4,3 millions), les maladies diarrhéiques (2,9 millions), la mortalité périnatale (2,4 millions), les pneumopathies chroniques obstructives (2,2 millions), la tuberculose (2 millions), la rougeole (1,1 million), les accidents de la circulation (1 million) et le cancer du poumon (0,9 million) [4]. En 2001, [5] on note une hausse des causes cardiovasculaires et une progression des traumatismes et des violences surtout dans la population adulte. Or les dépenses en santé restaient constantes sur les infections et les maladies transmissibles en général. Les projections par modélisation [6] montraient une progression de trois places des accidents de la circulation, de la 9^ème^ place à la 6^ème^ place, dans le top 10 des causes de décès dans le monde. Cette progression se faisait surtout dans les pays à faible revenu. Ces projections corrélées à l'analphabétisme expliquent la progression des traumatismes et des violences en Afrique. Selon ces projections, les traumatismes pointeraient à la 3^ème^ place des dépenses de santé à l'horizon 2020. Une autre méthode de modélisation de type « Diamond » [7] classait les accidents dus aux véhicules à moteur comme 4^ème^ priorité. Mais dès qu'on prenait en compte les inégalités dans le monde, ces mêmes accidents devenaient 2^ème^ priorité de santé. C'est pourquoi nous recommandons au ministère chargé de la sécurité routière et de transport de veiller à une bonne prévention routière par le port des casques pour les motocyclistes, l'utilisation effective des ceintures de sécurité, l'application stricte de la législation en matière d'alcool au volant, le contrôle des normes de sécurité des véhicules, la limitation de vitesse sur les voies rapides, la formation continue des usagers et l'introduction du permis à points. Le ministère de la santé devra continuer les efforts sur la prévention et la prise en charge des maladies transmissibles. Les deux priorités devront être les maladies cardiovasculaires et les traumatismes. La prévention des maladies cardiovasculaires notamment par la lutte contre les facteurs de risque cardiovasculaire doit être un effort national. Dans la prévention secondaire, la mise en place de défibrillateurs et l'éducation des populations au secourisme sont fondamentales dans ce domaine. Pour les traumatismes, le maillage adéquat du territoire en structures bien équipées et un système de ramassage efficient des blessés amélioreraient les chiffres de mortalité. De nombreux décès pourraient être évités par des mesures de prévention et de réglementation adaptées.

## Conclusion

L'étude des causes de décès dans deux hôpitaux représentatifs de Dakar montre la progression des décès par des causes cardiovasculaires et des traumatismes. Ce constat devrait induire une réorientation des ressources en santé vers ces domaines. Les maladies transmissibles restent importantes dans nos pays mais l'amélioration des conditions de vie induit une réduction de ces dernières. Un accent devra être mis sur la prévention des maladies de surcharge et leur prise en charge adéquate sur la voie publique et dans les structures de santé sur tout le territoire national. Les actions pour la réduction de la mortalité liée aux traumatismes devront agir sur le milieu, l'usager et les véhicules.

### Etat des connaissances actuelles sur le sujet

- Les causes de décès sont mal connues en Afrique sub-saharienne;
- La prédominance masculine parmi les décès;
- La prépondérance des causes cardiovasculaires et infectieuses.

### Contribution de notre étude à la connaissance

- Le maintien des causes cardiovasculaires comme première cause de décès;
- Le recul des causes infectieuses;
- La progression des traumatismes et plus généralement des morts violentes comme cause de décès.

## Conflits d'intérêts

Les auteurs ne déclarent aucun conflit d'intérêts.

## References

[1] Pierre Rouffet, Rachid Dekkak, Jean louis Solet, Bertrand Thélot, Philippe Morbidelli, Thomas Bastard (2009). Mortalité par accidents de la vie courante, île de La Réunion 2000-2004. Bulletin épidémiologique hebdomadaire.

[2] Naumann RB, Dellinger AM, Zaloshnja E, Lawrence BA, Miller TR (2010). Incidence and total lifetime costs of motor vehicle-related fatal and nonfatal injury by road user type, United States, 2005. Traffic Inj Prev.

[3] Guyavarch E, Pison G, Duthé G, Marra A, Chippaux J-P (2010). La mortalité violente dans trois régions rurales du Sénégal. Eur J Popul.

[4] Murray CJ, Lopez AD (1997). Mortality by cause for eight regions of the world: global burden of disease study. Lancet.

[5] Lopez AD, Mathers CD, Ezzati M, Jamison DT, Murray CJ (2006). Global and regional burden of disease and risk factors, 2001: systematic analysis of population health data. Lancet.

[6] Murray CJ, Lopez AD (1997). Alternative projections of mortality and disability by cause 1990-2020: Global Burden of Disease Study. Lancet.

[7] Lu TH, Huang YT, Chiang TL (2011). Using the diamond model to prioritize 30 causes of death by considering both the level of and inequality in mortality. Health Policy.

